# Editorial: Platelets and their multi-faceted roles in health and disease

**DOI:** 10.3389/fmolb.2024.1375090

**Published:** 2024-01-31

**Authors:** Marta Zarà, Gianni Francesco Guidetti

**Affiliations:** ^1^ Unit of Brain-Heart Axis, Centro Cardiologico Monzino IRCCS, Milan, Italy; ^2^ Department of Biology and Biotechnology, University of Pavia, Pavia, Italy

**Keywords:** platelets, hemostasis, thrombosis, biomarkers, signaling pathways

## Introduction

Blood platelets are commonly associated with the hemostatic response, as they are the main cellular component of the machinery that prevents bleeding. Understanding platelet function is essential to tackle different bleeding conditions and platelet transfusion represents a life-saving treatment for bleeding patients. Platelet are central also in thrombotic disorders and represent major therapeutic targets for these conditions. Therefore, most investigations in the field are focused on understanding the mechanisms of platelet activation for the identification of molecular targets to prevent thrombosis. Recently, novel roles have emerged for platelets, confirming their multifunctional nature. Already recognized physiological processes that rely on platelet function are inflammation and immunity, and platelets are well known players in several diseases, such as cancer, microbial infection and neurodegenerative disorders.

This Research Topic gathered six manuscripts which provide new insights on the complexity of platelets and their impact on human health.

## Platelet transfusion

Platelet transfusion is a life-saving practice used to treat hemorrhagic conditions, heavily bleeding trauma patients or oncohematological patients. The need for a continuous supply of platelets represents a challenge due to the limited shelf life of blood components. In this frame, cryopreserved platelets represent a worthy alternative, but their application has been tested in limited contexts (Noorman et al., 2016; Cohn et al., 2017; Bohonek et al., 2019).

Wikman et al. presented a prospective study involving adult bleeding patients with indication for platelet transfusion, to evaluate the use of cryopreserved platelets in remote hospitals which usually have limited platelet inventory. This investigation represents an important proof of concept and allowed the authors to conclude that the use of cryopreserved platelets in remote hospitals is feasible and increases the safety for bleeding patients in the cases where alternative approaches may be strongly delayed. Cryopreserved platelets can be potentially adopted as a backup stock also in larger hospitals experiencing acute shortage due to unpredictable causes. The authors also suggested that the procedure of freezing and thawing may be further improved to reduce the preparation effort and extend the storage time limit.

## Platelet alteration in pathology and their potential use as disease biomarkers

Mitochondrial dysfunctions have central roles in several common disorders, including cardiovascular diseases (Yang et al., 2022). The assessment of the bioenergetic profile of platelets has recently emerged as an interesting novel research field. Recent studies on platelet respiration support the concept that the bioenergetic function in circulating platelets may reflect the mitochondrial signature of other metabolically active tissues (brain, heart, liver, skeletal muscle). Therefore, alterations in mitochondrial respiration of platelets might have potential clinical applicability as a new diagnostic/prognostic tool.

Diaz et al. evaluated the association of platelet mitochondrial function with cardiometabolic health in a subset of 91 children (7–10 years old) enrolled in the Arkansas Active Kids Study. The study involved the use of high resolution respirometry to determine the association of platelet mitochondrial function with cardiometabolic health, evaluated in terms of adiposity, low-density lipoprotein and triglyceride levels, HOMA2-IR (marker of insulin resistance), cardiorespiratory fitness (peak VO2), and blood pressure. The authors provided convincing evidence that platelet bioenergetic in children correlates with their whole-body metabolic status. Interestingly, platelet mitochondria function did not associate with insulin resistance, but positively correlated with adiposity, high blood pressure, and peak VO2 and negatively correlated to dyslipidemia, confirming the link between mitochondrial function and clinical markers of cardiovascular health of children.

Abnormal platelet parameters have been detected also in autoimmune thyroid diseases (AITD). AITD is the most prevalent group of autoimmune disorders, and includes Graves’ disease (GD) and Hashimoto’s thyroiditis (HT). Altered platelet profiles (i.e., platelet-lymphocyte aggregates, thrombocytopenia, thrombocytosis) observed in patients with AITD, suggested that platelets may be involved in the pathogenesis of the disease (Tomczyńska et al., 2018). To deepen the understanding of the changes in platelet parameters in AITD, Cao et al. performed a meta-analysis including a total of 19 articles with 6,173 individuals (3824 AITD patients and 2,349 healthy people). Platelet count and mean platelet volume (MPV) values were significantly increased in AITD patients when compared with healthy controls, whereas no significant differences were found in platelet distribution width (PDW). Subgroup analysis based on disease type and thyroid function revealed that platelet count was significantly altered only in HT and hypothyroid groups. Conversely, MPV was significantly higher in GD patients and hyperthyroid and euthyroid groups. Although these results need detailed clinical studies to be validated, they confirm the correlation between platelet parameters and AITD that, in future, may provide broader clinical applications.

Abdominal aortic aneurysm (AAA) is characterized by the progressive dilatation of the abdominal aorta and inflammation-dependent degradation and remodeling of the vessel wall. Metz et al. have investigated the contribution of platelets in the progression of AAA focusing on Pannexin-1 (Panx-1), a member of anion-selective channels family involved in non-vesicular ATP release. In their work, the authors demonstrated that Panx1 plasma levels are increased in AAA patients, whereas platelet Panx1 tyrosine phosphorylation was reduced. In pancreatic porcine elastase (PPE) preclinical model of AAA, platelet Panx1 channels were found to be important for platelet activation, pro-coagulant activity and platelet-mediated inflammation. However, ECM remodeling and wall thickness, as well as aortic diameter expansion upon PPE surgery, were not changed in platelet-specific Panx1 deficient mice. In conclusion, the study pointed to a potentially important role for platelet Panx1 channels in inflammatory responses in AAA and adds important knowledge about the significance of platelets in AAA pathology.

The relationship between Alzheimer’s disease (AD) and platelets is complex and still largely unknown. AD is the most common dementia affecting elderly people and it is characterized by the development of amyloid plaques and neurofibrillary tau tangles in the brain, associated to neuroinflammation, neuronal death and cerebrovascular dysfunction (Cortes-Canteli and Iadecola, 2020). Through the years, several AD-specific platelet alterations have been described and their possible use as biomarker has been suggested.

The study by de Sousa et al. was aimed at understanding the evolution of platelet molecular profile, in terms of proteome and transcriptome, and the responsiveness of platelets with age and with the onset of AD. The proteomic signature of AD and aged individuals showed an increased platelet activation compared to controls. The analysis of mRNAs indicated the dysregulation of the ubiquitin-proteasome protein degradation system in platelets from AD, whereas the machinery mediating autophagy was altered in platelets from aged, non-demented individuals. Globally, the investigation shows that aging and onset of AD is associated to changes in the proteome and transcriptome of platelets and is confirmative of the general evidence that aging is coupled to altered platelet function.

## Novel mechanisms of platelet activation

Platelet activation is a key step in hemostasis and thrombosis, therefore the identification of new molecular mechanisms involved in this process is of utmost importance for the development of new therapeutic targets. This Research Topic includes a molecular-based investigation by Reusswig et al. focused on the role of N-methyl-D-aspartate receptor (NMDAR) in platelet activation. NMDAR belongs to the ionotropic glutamate receptors family, recently involved in platelet Ca^2+^ signaling. The authors have here exploited a platelet-specific NMDAR knock-out murine model to investigate the contribution of this receptor to thrombus formation. NMDAR knock-out platelets displayed normal activation-induced Ca^2+^ store release but defective store-operated calcium entry (SOCE). Such defect was associated to reduced Src- and PKC-dependent signaling and reduced integrin αIIbβ3 activation. Accordingly, thrombus formation under flow on collagen-coated surface was found to be reduced and NMDAR knock-out mice were protected against arterial thrombosis, indicating NMDAR as a possible novel target for anti-platelet therapy in cardiovascular diseases.

## Conclusion

The articles in this Research Topic are good examples of the multi-faceted roles of platelets and are part of a growing body of evidence highlighting the importance of these cells in health and disease. Future studies will be essential to provide further insights the field, with the aim of fully exploiting platelets as reliable therapeutical targets and diagnostic tools.
